# Projecting Month of Birth for At-Risk Infants after Zika Virus Disease Outbreaks 

**DOI:** 10.3201/eid2205.160290

**Published:** 2016-05

**Authors:** Jennita Reefhuis, Suzanne M. Gilboa, Michael A. Johansson, Diana Valencia, Regina M. Simeone, Susan L. Hills, Kara Polen, Denise J. Jamieson, Lyle R. Petersen, Margaret A. Honein

**Affiliations:** Centers for Disease Control and Prevention, Atlanta, Georgia, USA (J. Reefhuis, S.M. Gilboa, M.A. Johansson, D. Valencia, R.M. Simeone, K. Polen, D.J. Jamieson, M.A. Honein);; Centers for Disease Control and Prevention, Fort Collins, Colorado, USA (S.L. Hills, L.R. Petersen)

**Keywords:** Zika virus, ZIKAV, pregnancy, microcephaly, birth defects, flavivirus, viruses, Brazil, vector-borne infections

## Abstract

The marked increase in infants born with microcephaly in Brazil after a 2015 outbreak of Zika virus (Zika virus) disease suggests an association between maternal Zika virus infection and congenital microcephaly. To project the timing of delivery of infants born to mothers infected during early pregnancy in 1 city in Bahia State, Brazil, we incorporated data on reported Zika virus disease cases and microcephaly cases into a graphical schematic of weekly birth cohorts. We projected that these births would occur through February 2016. Applying similar projections to a hypothetical location at which Zika virus transmission started in November, we projected that full-term infants at risk for Zika virus infection would be born during April–September 2016. We also developed a modifiable spreadsheet tool that public health officials and researchers can use for their countries to plan for deliveries of infants to women who were infected with Zika virus during different pregnancy trimesters.

## Abstract 2

A modifiable spreadsheet tool will enable health officials to plan for these births.

In May 2015, the World Health Organization (WHO) reported an outbreak of Zika virus (Zika virus) disease in Brazil (*1*). Zika virus is a single-stranded RNA virus spread primarily by *Aedes aegypti* mosquitoes; maternal–fetal transmission of Zika virus has been reported (*2*). Zika virus infection is asymptomatic in many patients; when clinical illness does occur, it is generally mild, with exanthematous rash, fever, conjunctivitis, or arthralgia (*3*). An association with Guillain-Barré syndrome is under investigation; on rare occasion, death of patients with chronic disease has been reported (*4*).

In October 2015, Brazil started to report higher than expected rates of microcephaly among infants born in the same states where Zika outbreaks had occurred several months before (*5*). Laboratory tests later confirmed Zika virus infection in several infants born with microcephaly, and several case series have reported that mothers who delivered an infant with microcephaly had experienced Zika symptoms during early pregnancy (*5*–*8*). Because of the potential link between Zika virus infection and microcephaly, on February 1, 2016, WHO declared a public health emergency of international concern (*9*,*10*).

As of February 26, 2016, WHO reported 31 countries and territories (*11*) in the Americas in which local vectorborne transmission of Zika virus was ongoing (*12*). With expanding local Zika virus transmission and the possible link between Zika virus infection during pregnancy and congenital microcephaly, projecting the effects of Zika virus infections for other countries and understanding the gestational time when risk is greatest are critical. As has spread through the Americas, questions have arisen about the remarkably high numbers of infants with microcephaly reported in Brazil and the absence of reported microcephaly cases in some other countries where transmission is high. To help answer these questions, assessment of the timing of Zika virus transmission and its relation to gestational week of pregnancy for the cohort of women who were pregnant during the outbreak is necessary. Our report illustrates the expected periods of exposure and weeks of delivery for the cohorts of pregnant women potentially infected with Zika virus during outbreaks in Bahia State, Brazil. Public health officials and researchers in areas with local Zika virus transmission could apply these methods to country-specific data to produce more precise models and predictions.

## Methods

**Figure 1:**
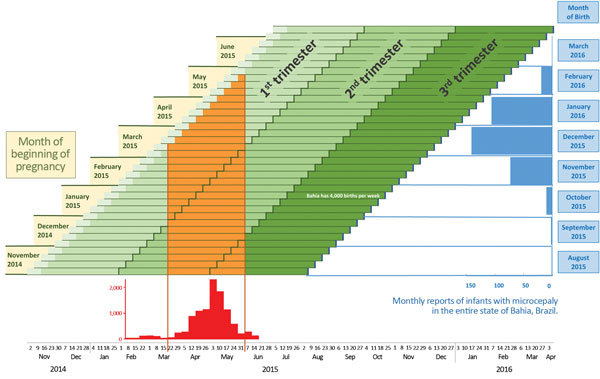
Projection of birth months after Zika virus transmission and occurrence of microcephaly, Salvador, Bahia State, Brazil. Weekly pregnancy cohorts are based on 40-week pregnancies and monthly reports of infants with microcephaly in Bahia State, Brazil, in relation to periods of high risk for Zika virus transmission. The epidemic curve shows cases treated for illness with rash in Salvadore, Brazil, estimated from (*14*). Complete monthly report data for January–March 2016 are not yet available.

Using published data for Bahia State and assuming that all pregnancies lasted 40 weeks (full term), we created figures demonstrating cohorts of pregnant women by week of delivery and then extrapolated to the beginning of pregnancy. Live-birth data from Brazil showed small differences in the proportions of infants born at full term (37–41 weeks) with microcephaly (76.7%) compared with those born at full term without birth defects (83.6%) (*13*). We considered the first 2 weeks of pregnancy to be the time from last menstrual period to conception (Figure 1). We also assumed the number of births to be constant across months of the year. To indicate the probable high-risk period for Zika virus transmission, we graphed the number of reported cases of Zika disease or Zika-like illness by epidemiologic week (the standardized method to enable comparison of weeks across years). We also graphed the reported cases of microcephaly by month of report, assuming that the month of report reflected the month of birth (*15*).

In Bahia, ≈4,000 infants are born each week (*16*); therefore, each bar represents ≈4,000 pregnancies. We derived epidemiologic data from a published report on exanthematous illness in the city of Salvador, Bahia State, Brazil (*14*). We assumed that the epidemic curve of exanthematous illness was representative of the epidemic curve of Zika virus infection and that the epidemic curve for the city of Salvador could be extrapolated to Bahia State. Because exact numbers of cases were not available, we derived estimates from the published epidemic curve, which was sufficient to identify the period of high Zika activity as being from March through June 2015. From the Live Birth Information System in Brazil (*16*), we obtained the monthly reports of infants born with microcephaly during August 2015–February 2016; information on births from January 2016 on were probably incomplete or were not yet available. The expected baseline prevalence of microcephaly is 6 cases per 10,000 births; for a state with 16,000 births per month, 10 cases of microcephaly would be expected each month.

**Figure 2:**
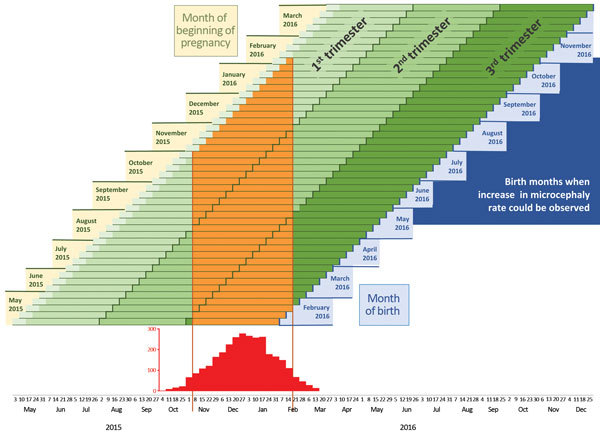
Projection of anticipated birth months after Zika virus transmission in a hypothetical country. Projected birth months for weekly pregnancy cohorts are based on 40-week pregnancies in a hypothetical country in which the highest level of Zika activity was from November 2015 through mid-February 2016.

To project the probable timing of births with adverse effects associated with Zika virus infection in early pregnancy, we then applied this approach to a hypothetical country. We assumed that transmission in Country A began on October 4, 2015, and followed the patterns that were seen in Salvador (*14*) and Yap Island (*3*). That is, we assumed that the level of transmission during October was low, during early November 2015 through mid-February 2016 was high, and from mid-February through mid-March 2016 was lower (Figure 2). 

## Results

In the city of Salvador, Zika virus transmission was highest during March–June 2015 (Figure 1) (*14*). During this period, a cohort of pregnant women could have been infected, and these infections would have occurred at different times during their pregnancies. The period of highest Zika activity was March 22–May 31, 2015 (Figure 1) across all cohorts. Pregnancies that began during November 2014–June 2015 correspond to births anticipated during August 2015–March 2016. For pregnancies that began in December 2014 or January 2015, the highest likelihood of Zika virus infection would have been late in the first trimester or during the second trimester of pregnancy, and these pregnancies would have resulted in term births during September and October 2015. For pregnancies that began during late February 2015–May 2015, the highest likelihood of Zika virus infection would have been during the first trimester, and term births would have occurred during November 2015–February 2016.

The increased number of reported cases of microcephaly in Bahia State began with October births; reported cases rose sharply during November 2015–January 2016. For the city of Salvador, these November 2015–January 2016 births corresponded to the highest likelihood of infection occurring in the first trimester or early in the second trimester of pregnancy, assuming that the date of report approximates the date of birth. There are no reports to indicate whether the city of Salvador experienced the Zika virus disease outbreak earlier or later than the rest of Bahia State.

In Country A (Figure 2), for the cohort of women whose pregnancies began in May 2015, corresponding to births during February–early March 2016, the likelihood of Zika virus infection would have been limited to the third trimester of pregnancy. Women whose pregnancies began in July 2015 would be expected to deliver in late March and early April 2016, and risk for infection would have been highest during the second trimester. The highest likelihood of first trimester and early second trimester infection would be among women who became pregnant during September 2015–January 2016, which corresponds to births from mid-May through early October 2016. 

To enable readers to project months when births with exposure in different trimesters can be expected, we developed a modifiable spreadsheet tool (Technical Appendix). Users may enter start and end dates of hypothetical outbreaks.

## Discussion

Our projections, based on ecologic data, indicate that in Bahia State, Brazil, Zika virus infection during the first trimester or early in the second trimester of pregnancy is temporally associated with the observed increase in infants born with microcephaly; this projection is consistent with the observed reported decline for January and February 2016. This finding adds to pathologic findings documenting Zika virus infection in several infants with microcephaly (*7*,*8*). To create a more precise projection of when to expect the first full-term births to mothers who were infected during their second trimester of pregnancy, readers can refine our model by using our modified spreadsheet tool (Technical Appendix) and local data from countries in which Zika virus is transmitted. 

Understanding the timing of Zika virus infection of pregnant women is key because the effects of infection on pregnancy and fetal and infant outcomes is likely to vary by gestational timing, as has been demonstrated for other congenital infections such as rubella and cytomegalovirus; transmission risk may also vary according to gestational timing (*17**,**18*). For rubella, risk for adverse fetal effects is highest during the first trimester; for cytomegalovirus, risk is highest during the first trimester but is also present after exposure during the second or third trimesters (*17*,*19*). For countries currently experiencing Zika disease outbreaks, it will be several months before the first pregnancies during which exposure could have occurred will reach term, particularly if the critical period of pregnancy is in the first or second trimester, as our data suggest.

Our hypothetical data (Figure 2) demonstrate the time between high levels of Zika virus transmission during pregnancy and pregnancy outcomes for each weekly cohort of pregnant women. With some shifting of dates, these projections could apply to many countries in South and Central America that are currently experiencing outbreaks of Zika virus disease.

We found ecologic evidence of a temporal relationship between maternal Zika virus infection during pregnancy and congenital microcephaly in Bahia State and the possible gestational time when risk is highest (Figure 1). This relationship does not necessarily imply causality, but it does give additional credence to the pathological findings and case reports that suggest a link between Zika virus infection and microcephaly (*1*,*5*). Assessing this relationship in other states in Brazil or other locations would have been informative, but very limited data on the spread of Zika virus are available. One limitation of the projections was that the estimated Zika virus epidemic curve for Bahia State was based on Salvador, the capital city, which contains only ≈18% of the population of Bahia State. It is unknown whether the timing of the outbreak in Salvador was similar to that in the remainder of the state, which served as the basis for the microcephaly case numbers. Also, the epidemic curve for Zika virus disease is not based solely on laboratory-confirmed cases, but rather it includes both suspected and confirmed Zika virus cases determined primarily on the basis of clinical presentation. The microcephaly data probably include some reporting delays, especially for January and February. Moreover, these projections assume a true association between maternal Zika virus infection and infant microcephaly; other maternal cofactors, such as other infections or environmental exposures, might account for some or all of the observed temporal relationship. The effects of the imprecision of some of the factors just described are unknown. Countries that can repeat this exercise with more precise prospective data will be better able to describe the expected critical exposure window, and if risk estimates for outcomes such as microcephaly and Guillain-Barré syndrome after Zika virus infection become available, the expected number of individuals who will be affected during a certain period can be predicted.

Some of the reported cases of microcephaly included in the graph are still being assessed, and some might not meet the final case definition for microcephaly in Brazil (i.e., head circumference <32 cm) (*20*); increased attention to the possible association between Zika virus infection and microcephaly may have led to overascertainment. However, the rate of false-positive reports was lower in Bahia than in other states in Brazil (*21*). Data on births of infants with microcephaly were available for September 2015–February 2016, and although the data from January and February 2016 are probably not complete, they do show a decline in the number of infants born with microcephaly. Maternal–fetal transmission might result in other adverse pregnancy outcomes, and the full range of these outcomes is of interest; however, our study accounts for microcephaly only. Also, our assumption of 40-week pregnancies does not account for possible differences in gestational age or for fetal losses and miscarriages, although early case reports do not indicate high rates of prematurity (*5*). If infants with microcephaly were consistently born premature, the relevant exposure period would be delayed to include more of the second trimester.

We assumed that the birth rates in these models remain constant throughout the year, which is not true for all locations. The data for Zika virus infection and infants with microcephaly are based on dates of report, which are probably later than actual occurrence.

Despite these limitations, our assessments provide some indication that the period of highest risk might be during the first trimester or early in the second trimester of pregnancy. This assessment can help inform public health officials about risks for microcephaly and help them plan for deliveries in areas where Zika virus disease outbreaks occur. Conducting surveillance for microcephaly but also other pregnancy outcomes such as pregnancy loss and other birth defects will enable continued evaluation of any effects of Zika virus disease might have on pregnancy. These data also emphasize the role of arboviral disease–tracking activities for informing public health planning. The US Centers for Disease Control and Prevention has prepared interim guidelines for US healthcare providers who care for women who are pregnant during a Zika outbreak (*22*) as well as interim guidelines for the evaluation and testing of infants whose mothers might have been infected with Zika virus during pregnancy (*23*). 

The consequences of Zika virus infection during pregnancy are not fully understood. Given the growing evidence of an association with microcephaly (*5*,*7*,*8*), and accounting for the time lapse between disease outbreaks and the birth of any affected infants as highlighted here, it can be expected that the number of infants born with microcephaly and other adverse pregnancy outcomes will continue to rise.

Technical Appendix Modifiable spreadsheet for projecting periods of delivery of at-risk infants after Zika virus disease outbreaks.
